# Podophyl. and Borax—In Diarrhœa

**Published:** 1881-09

**Authors:** E. B. Nash

**Affiliations:** Cortland, N. Y.


### CLINICAL BUREAU.

### Podoph. and Borax—In Diarrhœa.

E. B. Nash, M. D., Cortland, N. Y.

A child, one year old, but large for its age, with light complexion, black eyes, and dark hair, had diarrhœa for two months. In the beginning of the sickness, *Cham.* was given, on account of the great restlessness, with temporary benefit. A little later, *Calc. carb.* was given, on account of the leuco-phlegmatic temperament, large head, light-colored stools and crossness, but with no permanent benefit. Child was carried to the "Thousand Islands," in hope of benefit from the climatic change, but the benefit was only temporary, and the child was brought home as sick as ever, and weaker. At this time the following symptoms were elicited: Light-colored, offensive diarrhœa, containing undigested matter, stools more frequent in the morning and forenoon. Great restlessness and prostration; has not slept more than half an hour at a time, at night, for weeks. Grinding the teeth (the few that are out) and gums almost continually. Not much swelling of gums, but don't want them touched. *Appetite gone.* Gave *Podophyl.* CM. (Fincke) in solution, a teaspoonful after each evacuation. Only two doses were necessary, after which the discharge became natural, restlessness ceased, cheeks (before pale and sunken) flushed, appetite restored, sleeps at night. Cured.

B——, aged two months, has diarrhœa of greenish stools, every hour or two, day and night; cries much; mouth very sore; white coat on tongue and inside the cheeks. Mother has treated it with powdered borax and sugar, for several days; grew worse. As child has had borax without benefit, I gave *Cham.* and followed it some days after with *Sulph.* Grew worse fast, getting weak and emaciated; noticed that when the mother accidentally made a downward motion, with the child in her arms, it threw up its hands and showed fear of the downward motion. On inquiring, found this had been the case, all the time. Still the child had never been dropped or hurt by any falling! The child was very nervous, startling and crying at every sound; there was a red eruption on face; mouth was very sore, easily bleeding, raw especially under the tongue. *Borax* must be the remedy. Gave it in the 200th, dry on tongue; cured in a few days.

Thus we see that though borax in crude doses failed, after ample trial, the same given potentized in the minimum dose cured, as does always the *simillimum* remedy in proper dose. The other remedies failed because they were not homœopathic to the symptoms.

